# Design and Evaluation of a Collective Preventive Program for Musical Performance Anxiety (*ConfiDance*)

**DOI:** 10.3390/ejihpe14050083

**Published:** 2024-05-06

**Authors:** Belén Gómez-López, Roberto Sánchez-Cabrero

**Affiliations:** Department of Evolutive and Education Psychology, Universidad Autónoma de Madrid, 28049 Madrid, Spain

**Keywords:** musical performance anxiety, stage fright, musicians, musical interpretation, preventive program

## Abstract

Musical performance anxiety (MPA) is considered a subtype of social phobia and affects musicians who must face musical exposure in public, potentially severely affecting their emotional stability and significantly impairing the quality of their performance. This research has utilized previous scientific knowledge on the issue and a qualitative approach to musicians’ needs through focus groups in order to design a collective preventive program for MPA that could be implemented within the training curriculum of professional musicians. To evaluate the adequacy of the preventive program ‘*ConfiDance*’, a pilot test was conducted with a sample of 17 professional musicians in training, all post-graduate students in classical music performance. For the pilot test, a quasi-experimental model with a repeated measures methodology (pre-post and one-year follow-up after application) was carried out. The results indicate a significant decrease in MPA after the program implementation, with a notable improvement in effect one year post-application, demonstrating an even greater positive impact over time. These data should be interpreted cautiously due to sample limitations but represent an opportunity for the future implementation of a program that can prevent and treat MPA in music education centers.

## 1. Introduction

Musical performance anxiety (MPA) is a psychopathology classified by the World Health Organization [1] as a specific phobia. The American Psychiatric Association [2], on the other hand, defines it psychopathologically as one of the subtypes of social anxiety. It mainly affects individuals facing musical exposure in public, potentially severely compromising their performance or recital [3]. Performance-related anxiety constitutes an essential facet of musical execution and carries within it an adaptive function in the face of public exposure [4]. Debilitating forms thereof, referred to here, occur when physical, cognitive, psychological, or behavioral symptoms increase in intensity and persistence [4], significantly impacting all creative aspects and even mental and/or physical health [5].

The level of anxiety activation varies depending on the presence and correlation of multiple factors, such as the musician’s sensitivity to MPA during musical execution, the competence of the performer, the characteristics of the performance environment, the presence and characteristics of the audience, etc. [6,7,8]. In the case of music students, anxiety about performing at the highest level can become extremely intense, particularly in contexts where their performance is evaluated [9]. Thus, 20% of students who decide to abandon their musical careers do so for this reason [10]. Research in this regard points to the highly negative impact on musical execution and expresses the need for assistance in addressing associated problems [11,12,13,14].

### 1.1. Incidence

The experience of debilitating forms of MPA is common among both professional and student musicians. According to the findings of Ballester, who conducted a study with 570 participants, one in three musicians suffers from MPA [10,15]. These data align with those reported by Fernholz (2019), which place the prevalence rate of MPA among musicians between 16.5% and 60%, affecting approximately one-third of them (Fernandez-Granados, 2021). Additionally, according to Tamborrino [11], a significant percentage of professors (65%) and students (80%) from a prominent Midwestern university in the United States expressed the view that the prevention and reduction of performance anxiety should be taken into consideration in curriculum development of music programs [11,14].

The continuous presence of fear and high levels of anxiety can have detrimental effects on the psychological state and overall well-being of performers [9,16,17]. Derived from this issue and the instability of the lifestyle associated with their profession, musicians may experience a diverse range of mental disorders [9,18], adding a level of psychological vulnerability to this population.

### 1.2. Symptoms

The symptoms of MPA are grouped into four major categories [19]:

(a) Affective: characterized by apprehension and tension in musical performance situations [20,21,22].
(b) Somatic: such as alteration of heart rate and breathing, muscle tension, excessive sweating, etc. [23,24,25].
(c) Cognitive: such as lack of confidence in one’s abilities, anticipating catastrophic outcomes, setting unattainable goals, etc. [23].
(d) Behavioral: such as avoidance behaviors, substance abuse, etc. [23,26,27].

### 1.3. Age and Gender

It is noteworthy at this point that the prevalence of anxious symptomatology varies significantly among musicians according to sociodemographic variables. As to gender and age, women show a higher propensity to experience this phenomenon compared to men, and musicians over the age of 45 report a lower incidence of these symptoms compared to their younger counterparts [23]. This set of symptoms is linked to anxiety that arises at different stages of musical performance, from pre-performance preparation to post-performance evaluation [28,29], affecting both amateur and professional musicians [4,30,31].

### 1.4. Etiology

From a causal perspective, Barlow [32] proposes a triple vulnerability model: biological, general psychological, or specific psychological related to specific stimuli. This model will be adapted to the field of musicians by Kenny [33], who will redefine three more concrete factors: early relationships, individual psychological vulnerability, and personal concerns regarding public performance [4,34]. However, considering the multiplicity of factors influencing the origin of MPA, it must be conceptualized as a multidimensional and transactional phenomenon [35], avoiding where we should not attribute all responsibility solely to the individual and nor overlook the influences of the significant social and educational system components [36,37,38].

### 1.5. Therapeutic Approaches

The strategies commonly used by musicians to mitigate and cope with MPA symptoms include the use of prescribed beta-blockers or other non-prescribed medications, the consumption of antidepressants, anxiolytics or alcohol, increased instrumental practice, discussing the issue with the teacher or family, seeking assistance from a healthcare professional, whether mental or physical, relaxation and breathing techniques, hypnosis, familiarization with the concert venue, simulated stage practice, and positive self-talk (Burin, 2020). Of these techniques, those centered on emotions are the most commonly used by musicians, in contrast to problem-solving-focused strategies involving the action of external agents such as professionals and services, which are perceived as the most effective [39].

The quantity of research with sufficient methodological rigor on the treatment of MPA is notably limited [40,41,42]. Additionally, most of these studies incorporate therapeutic approaches that combine psychological and pharmacological treatments. However, the use of medication is discouraged in the treatment of MPA, as it interferes with exposure therapies and the disappearance of fear responses [14,43]. Furthermore, pharmacological action is limited to physical symptomatology, whereas the majority of MPA symptoms are emotional and cognitive in nature [14,33].

Some of the most scientifically explored psychotherapeutic interventions for addressing MPA are the following:

1. Cognitive-behavioral approach: widely studied; it trains individuals to recognize, examine, and modify harmful behaviors and thought patterns [4,44]. Grounded in the use of self-instructions and attention techniques, cognitive-behavioral therapy (CBT) addresses anxiety through stress management methods, such as specific problem-solving related to the instrument, mental techniques, breathing exercises, and relaxation training [4,45]. Exposure to virtual reality has also shown positive results [4,46].
2. Multimodal interventions: considered highly effective for addressing MPA, they amalgamate psychoanalytic therapies, cognitive-behavioral approaches, and methods of body awareness [4,30].
3. Body awareness techniques: including the Alexander technique [41], yoga [47], biofeedback technique [31], and meditation [4,48].

Other physio-behavioral techniques or psychotherapeutic approaches such as focusing on rhythmic breathing, visualization, or systematic desensitization have shown symptomatic improvements in MPA independently [34,40]. However, due to their contextual nature and replication challenges, these intervention strategies have not been fully incorporated into a specific program. Consequently, many of them currently lack the necessary scientific validation to assess their effectiveness [34].

The ability to deliver consistently high-quality performances under pressure emerges as a critical factor for the success and longevity of musicians’ careers [9,33,49]. Managing performance anxiety constitutes a continuous challenge, even for those performers with extensive experience in the musical field [9,50]. Therefore, it is essential to employ educational and training approaches that assist performers in preventing or, if necessary, intervening to prevent more significant problems once initial symptoms appear, thereby reducing the emotional and functional costs associated [51,52]. However, there are currently no preventive proposals for MPA implemented in educational settings with validated designs based on the scientific literature, as the primary focus of this field has primarily been on improving treatment once symptoms approach levels close to psychopathology [42,53]. These reasons prompted us to make this contribution, aimed at shedding some light on this important issue.

Specifically, this project aims to design a preventive program intended for collective application aimed at reducing or preventing the onset of anxiety symptoms associated with MPA in music students, strengthening their personal resources to cope with situations that threaten their professional development. The preventive program, designed based on scientific knowledge of MPA and its development, will be tested in a pilot study, evaluating its immediate and long-term effectiveness.

The hypothesis is proposed that the program will be effective in reducing anxiety levels, enhancing performance in live performance and individual study settings, as well as strengthening the musician’s relationship with music itself and the profession.

## 2. Materials and Methods

### 2.1. Design and Procedures

For this study, a quasi-experimental design without a control group and convenience sampling in a repeated measures design (pre-post with long-term follow-up) is utilized. To design the preventive program for MPA in music students in training, it is necessary, on the one hand, to ensure content validity by basing the selection of techniques and procedures on two sources of expert knowledge. Firstly, through an in-depth literature review on the subject in impactful academic databases and, secondly, through the assessment of an external expert committee [54]. On the other hand, an adaptation of contents adapted to the specific needs of music professionals in training, to whom an approach to this social reality was made through the application of focus groups.

Once the content of the proposed design is validated, a pilot study is conducted to evaluate its effectiveness in reducing MPA. For this purpose, pre- and post-measurements of MPA are carried out using the K-MPAI test [55]. Additionally, post-treatment MPA measurement is complemented with a qualitative evaluation of the program through a participant satisfaction questionnaire designed ad hoc (mixed design). Subsequently, one year after completing the program, participants undergo a re-assessment of MPA to evaluate the stability of the results obtained.

Finally, as the sample size is less than 30 experimental subjects and there is only one within-subject variable, the results are analyzed using the Friedman non-parametric test for repeated measures [56] and the Wilcoxon non-parametric test for related samples. Additionally, the extent of the effect is evaluated through partial eta squared. The relevance of the participant’s gender in the results and their previous personal history with MPA symptoms is also considered.

#### 2.1.1. Literature and Research Review

Initially, 71 research articles on MPA were identified in the major academic databases (Web of Science, Scopus, and Google Scholar) over the past five years [42]. Sixty-one of these articles were excluded, while ten of them were thoroughly analyzed. However, most of the studies found exhibited notable methodological deficiencies and sample limitations.

According to the review by Gómez-López and Sánchez-Cabrero [42], the treatments used in studies with positive results include (1) pharmacological treatments, such as the use of oxytocin, which improved the cognitive component of MPA, (2) traditional therapies within the field of psychology, such as Acceptance and Commitment Group Therapy (ACT) and its version of Acceptance and Commitment Coaching (ACC), (3) methodologies from Eastern philosophies such as mindfulness [51], (4) mind–body training combined with psychological therapies, cognitive-behavioral therapy exercises, mindfulness exercises, emotional regulation therapy, optimal experience states, and positive psychology, and (5) other proposals such as expressive writing.

Based on this review, the preventive proposal for MPA was designed, grounded in activities used in studies with positive results that could be carried out by personnel without access to pharmaceuticals or clinical therapies.

#### 2.1.2. Expert Committee and Informed Consent

Firstly, the overall study obtained approval from the IRB Scientific and Ethical Committee of the Autonomous University of Madrid (Spain), which reviewed the informed consent provided to participants and the detailed content of the pilot program (CEI-129-2648).

Subsequently, a committee of ten experts in the fields of music, psychology, and education, each with at least fifteen years of individual professional experience, evaluated the created preventive program. An ad hoc Likert scale questionnaire was developed to assess the competence and effectiveness of the proposal, as well as any potential blind spots and improvements to be implemented. Specifically, three classical music professionals, three modern music professionals, and four psychologists from the fields of clinical psychology and education were consulted. After applying the suggested changes, the test was resent to the same experts, who then approved the designed preventive program.

#### 2.1.3. Focus Groups

Given that it is a highly specialized and hard-to-reach population, we resorted to reviewing the institutions offering postgraduate studies in classical music performance in Madrid (Spain), both public and private. Finally, 16 postgraduate students in classical music interpretation from Alfonso X El Sabio University were selected. The participants, aged between 22 and 26, were chosen for their representativeness and willingness to participate. The discussion groups were divided by gender, with two groups comprising seven women and nine men, respectively. The sessions took place at the university during April 2023, were recorded in audio format, and lasted approximately one hour each. The discussion topics were based on a prior literature review and the consensus of four experts in classical music teaching. Specifically, the topics cited as initial guiding points were faculty, peers, pedagogical methods, and grades.

From the analysis of the discussion groups, several key points emerge. The figure of the teacher is of utmost importance to all participants, as they highly value and idealize it, considering it central to their educational development. They consider the pedagogical, emotional, and psychological training of teachers to be highly relevant for effective teaching, emphasizing the need for instruction beyond mere instrumental techniques and highlighting the lack of empathy and consideration for these dimensions in the classroom. In this regard, 43% of women have sought psychological help compared to 33% of men, although another 22% of the latter claim to believe they need it.

Both men and women agree that teaching methods in conservatories primarily focus on error, with little or no consideration for individual strengths or personal contributions to interpretation. There is a perceived rigidity in teaching methods, devoid of any enjoyment or development of personality or personal judgment. They narrate how being bound to accurately interpret the instructions on the score makes them feel restricted as artists and performers when being judged, lacking the personal space that musicians from other genres seem to have. As a result, the internal self-critical discourse when studying and performing is consistently present in a generic manner.

The promotion of rivalry among teachers and students within conservatories is also highlighted, fostering friendships based on competitiveness. This occurs more prominently with certain instruments, specifically violins, pianos, and cellos, resulting in these cases in a high degree of social pressure.

Women attach great importance to grades and identify them as a source of frustration, while for men, grades are a relatively minor concern, with greater importance placed on interpretation, enjoyment, and interpersonal interactions. In any case, both groups agree that their grades are often influenced by their relationship with the instructor and their individual circumstances.

Overall, the academic environment significantly influences the development of MPA, both through interactions with teachers and the values of excellence, competitiveness, and talent that govern the academic system. It is perceived as closed, elitist, competitive, inflexible, and prone to extremes, where music is considered a quasi-religious system, and the figure of the teacher is highly exalted [57,58].

These findings also confirm the trend of increasing hours dedicated to technical study without specific objectives [8,59]. Conversely, the results demonstrate that the effectiveness of the study is determined by its content and quality rather than the time invested in it [59,60].

Both groups express an almost total lack of preparation for stage performances in conservatories, as well as limited opportunities to perform as soloists, hindering their ability to become accustomed to public performances. Analysis of their discussions closely aligns with previous research highlighting the need for musicians to develop self-awareness and self-regulation tools in their academic and professional careers, which historically have focused predominantly on technical and interpretive aspects, neglecting the performer’s presence on stage [61,62].

The intervention proposals used in recent studies [42] encompass a variety of approaches, from traditional methods such as psychotherapy and cognitive-behavioral therapy [14,52] to treatments incorporating Eastern philosophies and mind–body training, either in conjunction with psychological therapies [34,62] or independently [4,51,63,64].

A prevention program should emphasize a range of techniques derived from these various approaches, which have been reviewed and adapted by a committee of experts in the fields of psychology, education, and music. Furthermore, the design of *ConfiDance* has been approached collectively, recognizing the significant amount of time typically spent in solitary study for MPA development. Sharing collective sentiments about the stage, relationships with peers, and perceptions of the music education system and the professional classical music world have proven to be contributing factors to its prevention in this investigation.

### 2.2. Population and Sample

After considering options for accessing musician populations for a pilot testing process, it was decided to limit the search to professionals in training who were pursuing specialized postgraduate studies in musical interpretation due to their proximity to professional dedication as musicians, either already being professional musicians or being very close to becoming one in the near future. Ultimately, the sample consisted of students enrolled in the Master’s program in classical music interpretation at Alfonso X El Sabio University in Madrid, Spain.

Due to the specificity of this population and the breadth and complexity of the designed program, only 17 participants between the ages of 22 and 26 voluntarily formed the final sample for the pilot test. However, it is a sample with a high level of specialization in a study of repeated measures, which does not require large population samples, and with profiles highly suitable for evaluating the effectiveness of the designed preventive program. Therefore, following the guidelines recommended by Onwuegbuzie and Collins [65], the research proceeded.

All of this made it impossible to conduct a high participation randomized intergroup investigation, and data on some variables, such as the impact of MPA by instrument, could not be concluded, given that some specialties had only one representative (bassoon, singing), others only had two (clarinet, violin, and cello), and three at best (trumpet, saxophone, and piano).

Of the seventeen participants, nine identified as male (52.9%) and eight as female (47.1%). Regarding their previous experience with MPA, eleven of them claimed to have experienced or currently experienced it (64.7%), while six of them (35.3%) claimed to have never experienced it.

### 2.3. Variables and Measurement Instruments

The evaluated variables and the tools to measure them were as follows:

#### 2.3.1. Independent Variable (IV): *ConfiDance*, Preventive Proposal for MPA

*ConfiDance* is configured as a collective preventive program for musical performance anxiety. The coined term *ConfiDance* combines *confidence* and *dance* to symbolize active harmony with confidence. This term suggests a dynamic and participatory sense of security, reflecting the notion of flowing with confidence as in a dance. The intervention consisted of a two-hour weekly session over eight weeks. These sessions were always held on the same day and at the same time to establish order, continuity, and commitment.

Activities were designed around three paradigms of interest defined by the techniques implemented in studies with positive results over the past five years [42]: (a) reflection and re-elaboration of thoughts and beliefs (activities linked to ACT and ACC), (b) body awareness exercises (mindfulness, yoga, breathing), and (c) activities triggering serotonin secretion (as pharmaceutical use was not available).

Within these three sets of interests, the following activities were carried out:

(a) Reflection and sharing on various topics were conducted, including why they chose to pursue music, the persistence of their initial drive, perceptions of themselves as music students and performers, self-esteem evaluation, assessment of instrumental and musical strengths, management of self-criticism, awareness of their judgments of other musicians, revisiting personal concepts of success, reevaluating career alternatives, and assessing the flexibility (or inflexibility) of their professional life plan. Additionally, changes in their experience during exposure and musical interpretation after the sessions were explored, along with highly detailed guided visualization of the complete process (before, during, and after) of a concert or audition, incorporating errors as part of the performer’s experience. Furthermore, negative thoughts were reframed into constructive ones in a positive and realistic manner, and thoughts and impressions were reviewed from the evaluator’s perspective as part of a fictitious tribunal, supplemented by expressive-reflexive writing.
(b) Breathing exercises (inhale for one count, exhale for double), apnea exercises to induce relaxation, mindfulness exercises (conscious breathing, awareness of thought flow, taking on the role of a passive observer without judgment), and yoga exercises (postures and sequences).
(c) Dramatic art exercises for emotional connection with musical form, playing music considered fun and connecting with positive sensations and/or memories (preferably, different from the usual study material), passages or pieces that are or have been part of the study repertoire after certain activities aimed at inhibition and dramatization, aiming to regain playful and expressive reconnection to playing, devoid of formal goals.

Furthermore, group dynamics of positive interpersonal interaction were conducted to facilitate group cohesion. These activities included cooperative, playful activities in two large groups, as well as others recommended by the expert group. These additional activities involved becoming aware of one’s escapes, engaging in limited free improvisation exercises initially with five notes on music distant from the classical genre, and then transitioning to pieces within the same genre. Participants also engaged in activities such as singing and dancing to performed works, creating a musical interpretation based on their biography, receiving positive feedback, and reflecting on how it was received, particularly if it came from fellow professionals. Additionally, participants explored improvised music using two possible scales to evoke images on different themes. They also practiced power postures for body awareness exercises inspired by Cuddy [66], reflected under pressure on their overall life plan, created flow maps to aid in the interpretation of a piece, and participated in exercises designed to raise awareness of their vulnerability and develop self-compassion.

The detailed schedule of specific activities, along with the temporal distribution of the sessions, can be found in Table 1 below.

Below, we categorize the activities according to their origin:

(a) Exercises used following the guidance of the expert committee: presentation and sharing/reflection (importance of fostering group cohesion and a trusting environment for sharing), theoretical introduction (providing support through scientific information to better understand this condition), awareness of personal escapes (practical work with this theoretical term), improvisation with five notes (without a formal objective, but playful and expressive), music autobiographical journey (using music for personal expressive purposes), guided music serving the image (limited free interpretation with a narrative purpose), mock tribunal (to experience firsthand the perception of a jury and what is judged by it), and flow map (to visually facilitate mental organization of the piece by zones and images).
(b) Research based on techniques of body awareness: body awareness/connection with the body (experiencing performance in contact with and through the body [34]), meditation [51,64], body relaxation and breathing exercises [34,62], and power posture exercises [66].
(c) Exercises derived from ACT (acceptance and commitment therapy), CBT (cognitive-behavioral therapy), emotional regulation therapy, and positive psychology: self-concept review as a musician and person [44,62], generally positive feedback and reflection [39,62], review of personal thoughts as an audience [4,39], awareness of negative automatic thoughts and limiting beliefs [14,30,52], thought reprocessing exercises [14,62], review of the concept of success [62], reassessment of life plan under pressure [67], exercises on vulnerability and self-compassion, and letter to oneself [14,62].
(d) Previous research based on serotonin treatments: laughter and well-being exercises to stimulate serotonin production (without medication) [28].
(e) Previous research based on visualization techniques: guided visualization to acclimate to aversive situations and normalize failure as part of performance [34,40,46].
(f) Previous research based on expressive writing [9].

#### 2.3.2. Dependent Variable (DV): Perceived MPA

Defined as a type of social anxiety in performance or public performance situations [4,40], MPA is characterized by a series of physiological, cognitive, and behavioral symptoms. In its debilitating forms, the experience of symptoms goes beyond a mere adaptive response, increasing their intensity and duration and potentially severely degrading musical interpretive skills.

To assess the level of MPA experienced by the participants, the Kenny music performance anxiety inventory (K-MPAI) [55] was used in its complete version, as it is the psychometric record shared by most studies in recent research. It is the only instrument developed for this purpose based on a psychological theory, Barlow’s triple vulnerability theory [32,68]. This self-assessment questionnaire, composed of 40 items, requires participants to rate on a Likert scale (0–6) the extent to which they experience various physiological, cognitive, behavioral, and emotional symptoms associated with MPA. Total scores range from 0 to 240, with higher scores reflecting higher levels of MPA. The K-MPAI is widely used in MPA research and has demonstrated outstanding internal reliability (Cronbach’s alpha = 0.94) (Chang-Arana et al., 2017), as well as convergent validity with widely accepted measures of trait and social anxiety [17,55]. According to Kenny [69], the cutoff point on the K-MPAI could depend on individual clinical issues of interest. In this study, the generally accepted clinical cutoff score of 105 was adopted [14,70,71,72].

Before the intervention, a semi-structured ad hoc interview was also implemented, in which participants were asked about personal aspects of their experience of MPA, in order to qualitatively evaluate the quantitative results obtained with the K-MPAI.

The administration of the K-MPAI questionnaire was repeated eleven months after the intervention to provide an extended follow-up over time. On this occasion, participants were once again provided with a semi-structured ad hoc interview to respond to questions about the impact of the program on their experience of MPA after these eleven months. The information was provided via email with forms created through Google Forms, and only seven out of the 17 participants responded.

#### 2.3.3. Within-Subject Intervening Variable: Evolution over Time

This is an ordinal within-subject variable with three levels: pre-treatment, post-treatment, and follow-up (11 months after the completion of the pilot study). Including this variable aims to assess the stability of the effect of the designed program once the initial momentum of the treated topics fades from participants’ recent memory, thus avoiding confusion between the effects caused by the intervention and those caused by experimental maturation, as reported in numerous similar previous studies [73,74].

#### 2.3.4. Between-Subject Intervening Variable: Gender

This is a nominal dichotomous attributive variable included to assess its relevance to the results of the independent variable.

#### 2.3.5. Between-Subject Intervening Variable: Participants’ Previous MPA History

This is a nominal dichotomous variable included to assess its relevance to the results of the independent variable, referring to a person’s past experiences related to anxiety experienced during public performances or performance situations. It includes events and situations in which the person has experienced MPA, as well as the frequency, intensity, and duration of these episodes over time. Additionally, it may include specific triggering factors, coping strategies used, and the impact that anxiety has had on the person’s personal, professional, or academic life [19].

**Figure 1:**
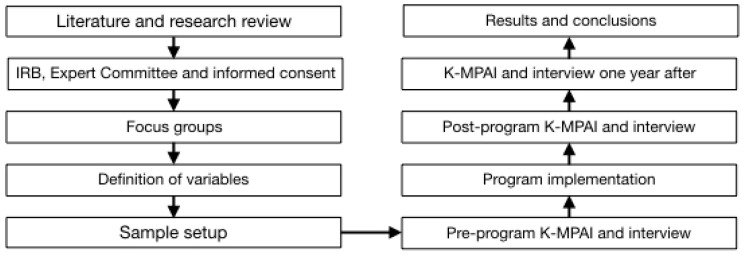
Steps of the research process.

Figure 1 below illustrates the steps of the research process.

## 3. Results

Below, Table 2 shows the frequencies and percentages of pilot study participants according to the considered attributive variables and their participation in the three MPA evaluation measures (pre-treatment, post-treatment, and long-term follow-up).

Table 2 reflects how the sample size implies considering the violation of the necessary conditions of homoscedasticity and normality for inferential statistical analyses, given that it is a small sample, especially taking into account its division among different conditions of the attributive variables (gender and previous MPA experience). However, a large sample is not necessary for a repeated measures study, especially if it is a very homogeneous sample that serves its purpose as a pilot study to empirically test the functionality of the designed preventive proposal, as it is a sample selected for its high incidence in problems related to MPA. It is also worth noting the high experimental mortality suffered in the long-term follow-up measurement, which reduces the total sample by 58.8%.

Next, Table 3 shows the descriptive statistics obtained in the pilot study through repeated measures using the K-MPAI test, considering the attributive variables under consideration.

**Figure 2:**
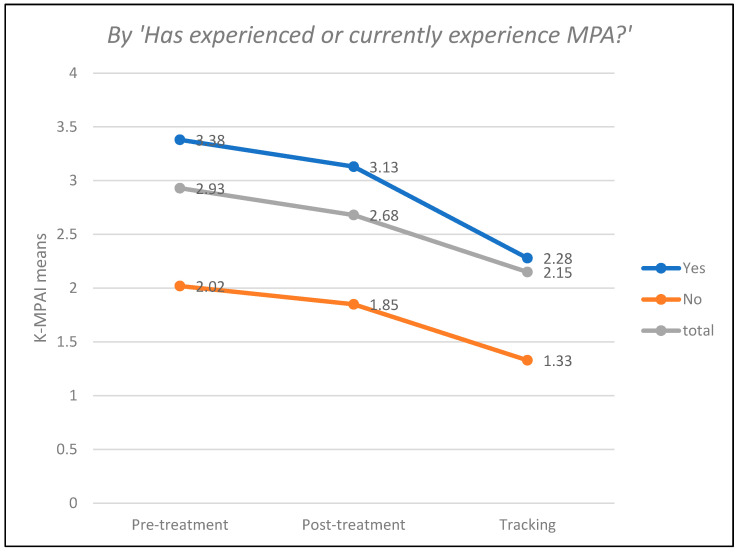
Results of the K-MPAI test by MPA experiences.

**Figure 3:**
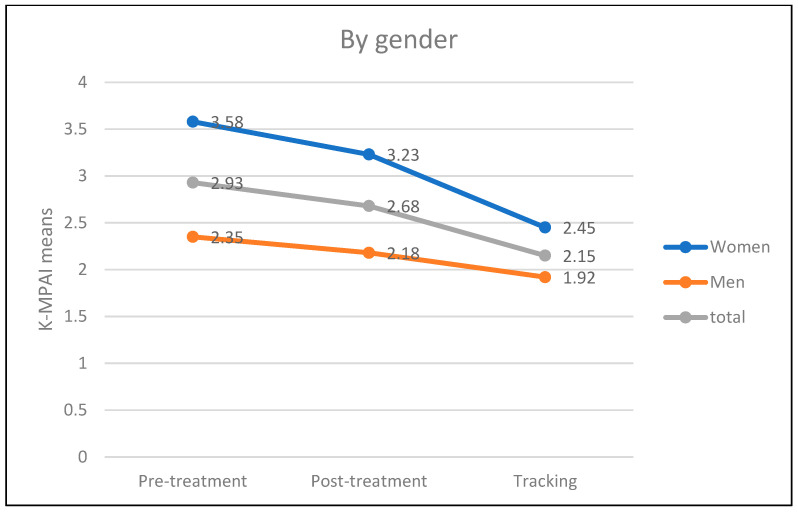
Results of the K-MPAI test by gender.

**Figure 4:**
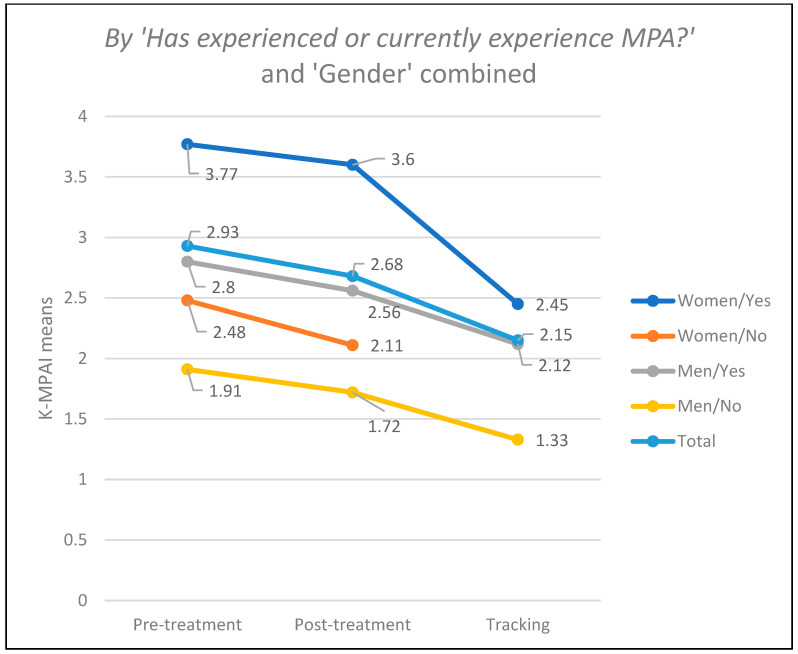
Results of the K-MPAI test by MPA experiences and gender combined.

In Table 3, a clear reduction in MPA, measured through the K-MPAI test, can be observed in the post-treatment and follow-up measurements compared to the pre-treatment measurement globally and under all conditions of the measured variables. However, some conditions of the considered variables have a reduced N to 1 subject or even 0, which discourages including these variables in inferential analyses, requires these results to be viewed with caution, and prohibits generalizations. Figure 2, Figure 3 and Figure 4 below visually show this reduction in MPA over time.

To evaluate the improvement produced by the implemented proposal in the pilot study (IV) on the participants in the study through the measurement of a single within-subject DV (K-MPAI test) in a repeated measures design with follow-up in a sample with less than 30 participants, the results obtained in the Friedman test and Wilcoxon signed-rank test for paired samples are used. Additionally, due to the very small sample size, including the extent of the effect measured through partial eta squared in repeated measures, ANOVA is advised, as shown in Table 4 below.

The results from Table 4 highlight how the IV (preventive proposal) leads to significant results in the DV (MPA measured through K-MPAI) that are maintained over the long term. In fact, they cause an effect that expands over time. That is, the effects of the designed proposal are more clearly seen in the follow-up measurement after twelve months than in an immediately subsequent measurement.

Regarding the degree of distress, this study has considered it important to take into account the level of intensity experienced by the participants, specifically in the case of those suffering from debilitating forms of MPA, as it is a determining factor for inferring conclusions about the effectiveness of the treatment. The data conclude, once again, a higher intensity of MPA in the case of women than men.

According to the results of the K-MPAI test, at the beginning of the research, 66.66% of the participants exceeded the thresholds of debilitating forms of MPA, of which 40% were women and 26.66% were men. The remaining 33.33% believed to suffer from MPA in an adaptive form or not to suffer from it at all. Of this latter percentage, 26.66% were men, and 6.67% were women. After the program application, the percentage of participants whose data were above the threshold of debilitating forms of MPA was reduced to 52.4%, and it was 28.57% eleven months after its completion.

Before commencing the program, qualitative data indicated that participants, particularly men, exhibited a higher perception of self-worth and self-esteem compared to women. Within the musical domain, a concerning scenario emerges regarding the mental health of most participants, with a greater prevalence of negative data among women, as previously noted. Most participants reported feeling undervalued, inadequately guided, emotionally neglected, isolated, and, in some instances, humiliated, with a pronounced emphasis on the relentless pursuit of technical perfection and stage confidence in competitive and hostile environments.

Regarding the qualitative findings from the semi-structured interview, only seven out of the 17 participants responded to the interview provided one year after the treatment. However, all of them stated that they had improved in some aspect of MPA management, feeling more confident, less concerned about others’ reactions, or simply more focused on playing. In addition, their attitude towards tests and auditions was more positive, resulting in feeling increasingly better and obtaining improved outcomes by managing to control their nerves without affecting their performances. All these data are consistent with the quantitative results obtained with the K-MPAI questionnaire.

The strategies that helped them, and that are still valid a year later, ranged from putting the situation and its risks into perspective, practicing breath-holding, studying with visualizations, and developing concepts discussed during the sessions. Some of the resources that were discussed and agreed upon included indulging in non-musical pastimes, playing for one’s enjoyment, not living so obsessively and toxically focused on studying the instrument, and testing in general. A common response from all those who answered the interview was how much openly discussing their feelings with other colleagues had helped by making them aware that their fellow students had similar fears and how they dealt with them.

## 4. Discussion

At a general level, the results obtained indicate that preventive intervention in MPA with professional musicians in training is very positive for preventing or reducing MPA symptoms, especially considering the great stability shown by the results. Therefore, the initial hypothesis would be confirmed. The data even indicate an improvement over time, suggesting that the discovery and mastery of therapeutic techniques introduced in this initial preventive intervention fostered awareness among participants and enhanced their application through ongoing involvement and practice [51,52]. This notion is echoed by one participant’s metaphorical depiction, describing how they planted a “seed” that gradually flourished over the months. This effect has already been seen in other therapeutic areas, as shown by recent studies such as Barrett and Stewart [75], who observed it in similar mindfulness-based techniques, or Sánchez-Cabrero [74] in the prevention of disorders related to body image.

The reviewed studies from the last five years implementing interventions for improving or reducing MPA [42] have revealed several limitations in their design, which this study has endeavored to control. The main weaknesses were the use of small samples, the absence of a control group and/or follow-up, the use of cognitive evaluations exclusively, and, in one case, the lack of prior validation of the intervention used. This research aimed to standardize data collection using the K-MPAI test, aligning itself with the majority of studies on MPA treatment. Thus, differences in the methods used for data collection and the heterogeneity of their designs make comparison and synthesis of their results problematic. The data were collected at different times during the research (before its application, immediately after its completion, and eleven months later) to ensure the reliability of the results. In addition, short-answer descriptive interviews were used to qualify the numerical data obtained. In this regard, the evaluations focus on self-perception data of cognitive, behavioral, and physiological processes. The collection of biological samples or the use of pharmacological treatments was not part of this study due to the professional limitations of the researchers in carrying out such activities [14,33]. The methodology used was based on previously researched and implemented techniques with positive results and on methodology reviewed and validated by an expert committee, as recommended by previous studies [14,39].

However, despite the very positive results obtained in this pilot study regarding the long-term benefits of implementing the proposal, the lack of a control group, as well as the small sample size, necessitate a cautious interpretation of the data as in previous research [53]. This is a factor that is repeated in most of the reviewed studies from recent years [42], and that calls into question large-scale conclusions about the effectiveness of the different proposed methods. In the case of this research, we have encountered the same problem concerning a large sample size, given the low number of annual enrollment in higher education centers for each musical instrument. Similarly, the sample comprises a social group consisting of young professionals in training between the ages of 22 and 26, which limits the generalization of the results to other age groups or professional situations. Other limitations to consider include the absence of control groups and the lack of blinding in the study scope. These features of the study design increase the risk of biases, such as observer bias.

Regarding the experience of MPA by gender, the results conclude that, as affirmed by previous research [6,33,55,76,77], women suffer from this type of anxiety in a higher proportion and with greater intensity than men. Specifically, in the phase prior to the implementation of the program, 85.7% of women showed clear episodes of MPA compared to 55.56% of men. In the phases following the program, both immediately after its completion and one year later, the data continue to maintain higher values in women than in men, although in both cases, the incidence of MPA decreases over time.

The relevance of gender as a mediating factor in the onset and development of MPA, observed in this study, has been identified previously in other research, such as that of Osborne and Kenny [78], Yoshie et al. [25], or more recently, Fernández-Granados (2021). According to these studies, gender differences are closely related to a greater cognitive predisposition and greater affective-emotional relevance in women [79,80]. In other words, the different social demands associated with female roles in Western society predispose women to be more sensitive to social criticism, which is directly related to a greater initial predisposition to MPA symptoms.

Conversely, the low participation rate in the tests and interviews one year after program implementation, along with the improvement in scores and favorable testimonials, may be attributed to higher participation in this latter phase by individuals who were highly satisfied and motivated with the program’s results, and thus more willing to engage in the research. It should also be noted that the initial data indicate that 11 out of the 17 participants were experiencing debilitating forms of MPA, which may explain why the six participants who did not suffer from it in its pathological form did not show the same interest in responding to the questionnaire one year later. Additionally, some of the personality traits of musicians may have also influenced certain participants’ willingness to engage in a follow-up interview, especially after a long period. Among the most significant are individualism, competitiveness, dichotomous thinking, self-criticism, diva behavior, perfectionism, and catastrophic thinking [81,82]. In this case, traits such as individualism may have generated disinterest after many months, while others, such as self-criticism or perfectionism, may have elicited reluctance or anxiety when facing their emotional experience again. In any case, the low participation in this final phase necessitates that the data regarding should be considered even more cautiously.

## 5. Conclusions

Upon reviewing the results of this program and the consequent long-term improvement in MPA symptoms, the benefits in terms of self-awareness development and strategies for the prevention and management of this psychopathology in the participants of the studied sample are confirmed.

Given the high percentage of musicians affected by this condition, both in the professional and academic worlds, the promotion and implementation of content addressing MPA at all levels of music education are revealed as essential in order to improve the mental health of this group. This concerns not only the focus on performance but also the need to train potential future professionals in music education from specialized educational institutions.

Therefore, the main strengths of this intervention lie in the potential benefits offered by the inclusion and implementation of a prevention program in music education centers commencing at a very early age. Consequently, this would favor the implementation of a standardized prevention program in professional conservatories, higher education institutions, or master’s programs, as well as in professional populations not in a training period. All these data provide us with the opportunity to meet the next objectives posed by the results of this study, primarily with respect to the refinement of the program, seeking new strategies for its large-scale implementation, thus addressing the limitations imposed by the sample size.

## Figures and Tables

**Table 1:** Summary of ConfiDance program activities.

Sessions	Activities per Session
Session 1	Presentation and sharing (20 min) Theoretical introduction to MPA (30 min) Awareness of personal escapes (20 min) Group free improvisation with five notes (15 min) Body awareness/connection with the body (singing/body). Option for individual volunteers (20 min) Sharing and reflection: group discussion (10 min) Weekly plan and closing: attention to escapes and avoidance strategies, expressive writing (5 min)
Session 2	Sharing (15 min) Apnea exercises (20 min) Group cohesion devoid of musical demands or formal objectives: cooperative activity (singing in small choral groups if not singers, small theatrical piece, etc.) (40 min) Self-concept review as a musician and person (40 min) Weekly plan and closure: reworking thoughts, expressive writing, meditation, and reflection (5 min)
Session 3	Sharing (15 min) Musical autobiographical journey (30 min) General positive feedback and reflection (30 min) Review of personal thoughts as an audience (20 min) Group reversal of these thoughts (20 min) Weekly plan and closure: awareness during the next week, expressive writing, meditation, and reflection (5 min)
Session 4	Sharing (15 min) Awareness of negative automatic thoughts and limiting beliefs (30 min) Thought reprocessing exercises (30 min) Laughter and well-being (serotonin) (10 min) Playful and limited performance: guided music serving the image (30 min) Weekly plan and closure: negative automatic thoughts reversal, expressive writing, meditation, and thought reversal (5 min)
Session 5	Sharing and discussion (15 min) Body relaxation and breathing exercises (15 min) Voluntary performance for enjoyment, unrelated to repertoire, style, or even the instrument of study (15 min) Guided visualization and voluntary performance (40 min) Positive feedback in small groups and reflection (30 min) Weekly plan and closure: expressive writing, meditation, and reflection (5 min)
Session 6	Sharing and discussion (15 min) Body awareness and power posture exercises (Amy Cuddy) (20 min) Review of the concept of success (40 min) Reassessment of life plan under pressure (40 min) Weekly plan and closure: expressive writing, meditation, and reflection (5 min)
Session 7	Sharing and individual preparation (15 min) Mock tribunal where participants act as both jurors and performers (90 min) Final analysis and conclusion (15 min)
Session 8	Group discussion (15 min) Exercises on vulnerability and self-compassion (20 min) Letter to oneself (15 min) Flow map (20 min) Voluntary performance (20 min) Final reflection and group discussion (30 min)

**Table 2:** Frequencies and percentages.

		Pre-Treatment			Post-Treatment			Tracking		
		Women	Men	Total	Women	Men	Total	Women	Men	Total
*Has experienced or currently experiences MPA*	*Yes*	6 (35.3%)	4 (23.5%)	10 (58.8%)	6 (35.3%)	5 (29.4%)	11 (64.7%)	3(17.6%)	3 (17.6%)	6 (35.3%)
	*No*	1 (2.7%)	4 (23.5%)	5 (29.4%)	2 (11.8%)	4 (23.5%)	6 (35.3%)	0 (0%)	1 (2.7%)	1 (2.7%)
TOTAL		7 (41.2%)	8 (47.1%)	15 (88.2%)	8 (47.1%)	9 (52.9%)	17 (100%)	3 (17.6%)	4 (23.5%)	7 (41.2%)

**Table 3:** Descriptive statistics.

		Pre-Treatment (Mean (SD))			Post-Treatment (Mean (SD))			Tracking (Mean (SD))		
		Women	Men	Total	Women	Men	Total	Women	Men	Total
*Has experienced or currently experiences MPA*	Yes	3.77 (0.79)	2.8 (0.56)	3.38 (0.84)	3.6 (0.92)	2.56 (0.55)	3.13 (0.94)	2.45 (0.59)	2.12 (0.8)	2.28 (0.65)
	No	2.48	1.91 (1.15)	2.02 (1.02)	2.11 (0.9)	1.72 (1.06)	1.85 (0.94)	-	1.33 (0)	1.33 (0)
TOTAL		3.58 (0.87)	2.35 (0.56)	2.93 (1.09)	3.23 (1.09)	2.18 (0.91)	2.68 (1.1)	2.45 (0.59)	1.92 (0.77)	2.15 (0.7)

**Table 4:** Inferential tests.

Contrasts	Chi-Square	p (Friedman Test)	p (t Wilcoxon Signed-Rank Test)	Partial eta Squared
Pre-post	1.667	0.197	0.125	0.176
Pre-tracking	3.571	0.059	0.028 *	0.715
Post-tracking	0.143	0.705	0.176	0.450
Pre-post-tracking	7.714	0.021 **	.	0.661

* Correlation significant at the 0.05 level/** Correlation significant at the 0.01 level.

## Data Availability

The specific contents of the *ConfiDance* activities are available for consultation upon specific request to researchers, as the right is for the publication of program-specific material reserved by researchers in the future.
